# Comparison of methods for recruiting and engaging parents in online interventions: study protocol for the Cry Baby infant sleep and settling program

**DOI:** 10.1186/s12887-015-0502-9

**Published:** 2015-11-10

**Authors:** Fallon Cook, Monique Seymour, Rebecca Giallo, Warren Cann, Jan M. Nicholson, Julie Green, Harriet Hiscock

**Affiliations:** Parenting Research Centre, 5/232 Victoria Parade, East Melbourne, 3002 VIC Australia; Murdoch Childrens Research Institute, Royal Children’s Hospital, Flemington Road, Parkville, 3052 VIC Australia; Judith Lumley Centre, La Trobe University, 215 Franklin St, Melbourne, 3000 VIC Australia; Centre for Community Child Health, The Royal Children’s Hospital, Flemington Road, Parkville, 3052 VIC Australia; Department of Paediatrics, University of Melbourne, Melbourne, Australia

**Keywords:** Infant, Sleep, Crying, Online, Intervention, Parents

## Abstract

**Background:**

Anticipatory guidance around the management of sleep and crying problems in early infancy has been shown to improve both infant behaviour and parent symptoms of postnatal depression. Digital technology offers platforms for making such programs widely available in a cost-efficient manner. However, it remains unclear who accesses online parenting advice and in particular, whether the parents who would most benefit are represented amongst users. It is also unknown whether the uptake of online programs can be improved by health professional recommendations, or whether parents require additional prompts and reminders to use the program. In this study we aim to: (1) determine whether weekly email prompts increase engagement with and use of a brief online program about infant sleeping and crying, (2) determine whether encouragement from a maternal and child health nurse promotes greater engagement with and use of the program, (3) examine who uses a brief online program about infant sleeping and crying; and, (4) examine the psychosocial characteristics of participants.

**Methods/Design:**

This study is a randomised, parallel group, superiority trial, with all participating primary carers of infants aged 2 to 12 weeks, receiving access to the online program. Two modes of recruitment will be compared: recruitment via an online notice published on a non-commercial, highly credible and evidence-based website for parents and carers and via the parent’s Maternal and Child Health nurse. After baseline assessment, parents will be randomised to one of two support conditions: online program alone or online program plus weekly email prompts. Follow up data will be collected at 4 months of infant age.

**Discussion:**

Results from this trial will indicate whether involvement from a health professional, and/or ongoing email contact is necessary to engage parents in a brief online intervention, and promote parental use of strategies suggested within the program. Results of this trial will inform the development of recruitment and engagement strategies for other online interventions.

**Trial registration:**

Australian New Zealand Clinical Trials Registry: ACTRN12613001098729. Registered 01 October 2013.

## Background

Infant sleep and crying are common concerns for parents of infants [1] with 12 to 27 % of parents reporting infant crying problems within the first 4 months [2, 3], and 16 to 38 % of parents reporting infant sleep problems within the first year [2–4]. Common parent concerns centre on the amount and pattern of their infant’s sleep, strategies for settling, and how to best manage infant crying [5]. Evidence suggests that anticipatory guidance delivered face-to-face, helps parents to establish good infant sleep habits, reduces parent distress about normal infant crying, and reduces postnatal depression (PND) symptoms [3, 6–10]. However, it has not been determined whether this type of approach can be effectively delivered online. The internet potentially offers a cost effective and convenient platform for delivery of anticipatory guidance to large and geographically diverse audiences. Currently, little is known about the characteristics of parents who particpate in online programs during the early postpartum period, or how to best enhance recruitment and maintain engagement with the program content. An evidence based program, *Cry Baby*, was developed for online delivery. The aim of this study is to examine the characteristics of parents (and their infants) who seek help for infant behaviour via a parenting website, and identify any under- or over- representation of population subgroups within the sample. We also seek to determine whether encouragement from a health professional to take part in an online program, and/or regular weekly email support, promotes greater engagement with the program and greater use of suggested strategies.

Infant sleep problems are common (38 % of parents of 4 week old infants report infant sleep problems) [3], costly to treat [11], and are associated with increased risk of PND [12] and parental fatigue [13]. A number of randomised controlled trials have shown that behavioural-education interventions that give advice on how to set up good sleep habits in infancy can successfully prevent problems with infant settling and night waking [3, 6–10]. These interventions promote parenting efficacy; enhancing parent confidence and competence with managing infant sleep and crying behaviours [10].

Infant crying is normal and has a natural peak in frequency of around 2.5 h of crying per day at around 6–8 weeks of age [1]. While infant crying rarely has a medical cause [14], 27 % of parents report their infant’s crying is a problem and many of these parents will seek assistance in the belief that something is medically wrong [2, 3]. Educating parents on normal infant crying behaviour may reduce parental distress and the incidence of parents unnecessarily seeking costly medical support. Additionally, providing parents with advice on strategies for coping with infant crying, may help prevent cases of Abusive Head Trauma (AHT; previously known as Shaken Baby Syndrome) [15]. Frequent infant crying is a proximal risk factor for AHT, and is potentially more readily modifiable than other risk factors such as being of low socioeconomic status, or born prematurely [16].

Mothers who report infant sleep and crying difficulties are at increased risk for PND [12, 13, 17–19]. In a short-term longitudinal study, Goldberg and colleagues [18] found that mothers who were experiencing symptoms of distress at 6 months had infants with more sleep and crying issues, and reported being more bothered by those issues. This trend was still apparent when the infant was 12 months of age. Hiscock and colleagues [20] found that providing parents with a brief behavioural intervention delivered by a primary healthcare professional at 8 months of infant age was effective in reducing infant sleep problems and improving maternal mental health. Early intervention for unsettled infant behaviour, may reduce the risk of ongoing maternal symptoms of depression and distress through later infancy. Emerging research also suggests that infant sleep and settling problems may impact on parental fatigue [13]. In an Australian sample Giallo and colleagues [13] found the mothers of young children (0–4 years) with sleep and settling difficulties had an increased risk of experiencing high levels of fatigue, with the potential for adverse impacts upon their daily functioning, parenting and later child outcomes.

Several research trials have established the effectiveness of interventions designed to improve infant sleep or help parents manage infant crying [7, 9, 10, 21, 22]. An Australian randomised controlled trial evaluated the *Baby Business* program, which provided anticipatory guidance on infant sleep and crying with content delivered via booklet, DVD, telephone consultation and a parent group session [3]. Compared to the control condition, the program resulted in significantly better outcomes for mothers and infants, including a greater decline in PND symptoms from 4 to 6 months post-natally, less time spent attending to the infant during the night, fewer changes to infant formula to ‘manage’ infant behaviour, less doubt about settling the infant at bedtime and better ability to set limits at bedtime. Additionally, mothers of a sub-group of infants that were ‘frequent feeders’ (fed greater than 11 times per 24 h) reported fewer daytime sleep and crying problems. These findings bode well for the prevention of future sleep problems in these infants.

While the efficacy of face-to-face interventions has been established, low income parents and those residing outside of major metropolitan areas, face major obstacles to accessing parenting interventions. Key barriers include difficulties with transportation, problems accessing childcare to attend appointments, and inflexible work hours [23]. Studies have shown that mothers of young children frequently consult the internet for information about their children’s health [24–26] and social isolation amongst young mothers is associated with greater amounts of time spent online overall [27]. A study from the Royal Childrens Hospital (Melbourne, Australia) indicated that 81 % of mothers had access to the internet and 18 % had changed the way they managed their children’s health based on information found online [28]. While the internet offers the potential for better access to evidence-based interventions for vulnerable, low income and/or remote families, particularly via the ubiquitous smartphone, little is known about actual uptake of online interventions by these families. Given the higher adoption rates of home internet connection and smartphone ownership in higher income groups [29], it is possible that online programs that target infant sleep and cry problems will attract the same over-representation of parents who are more highly educated and of higher socioeconomic status, as traditional face-to-face interventions [3].

One way this imbalance may be addressed is for infant health care providers to encourage participation. In the Australian state of Victoria all newborn infants are assigned a Maternal and Child Health (MCH) nurse, who provides free health checks at 7 to 10 days, 2 weeks, and then at 1, 2, 4, 8, 12, 18, 24 and 42 months of age. Access is almost universal – 98 % of infants participate in the first (in-home) visit, and 96.6 % attend the MCH clinic for the 2 week (infant age) check up [30]. MCH nurses are in a unique position to direct parents to suitable evidence-based online interventions that are specific to the first few weeks postpartum, because they have frequent contact with parents during this time. In addition, evidence suggests that support or encouragement from nurses or other health professionals during online program participation helps to keep parents engaged [31, 32]. A strategy often employed to increase engagement and retention to online programs, is the use of email prompts, however, little research has specifically examined their effectiveness. Email prompts have been shown to increase the number of adults who returned to computer tailored lifestyle interventions (targeting smoking behaviour and fruit and vegetable intake, for example) [33], and this effect was increased when the prompt occurred soon (2 weeks) after initial program login [34], but we can find no research that evaluates the usefulness of email prompts in samples of parents of infants, whose needs and time demands would be quite different. Uptake of, and retention to, online programs varies considerably, but is generally reported to be lower than anticipated (~2–10 % and ~15–97 %, respectively) [35–39]. If online interventions are intended to be used broadly, then evidence for strategies employed to engage and retain participants, will be necessary.

Research examining the reach and acceptability of online parenting programs is still in its infancy, however, results so far look promising. A trial of an online intervention designed to treat *existing* sleep problems in infants/toddlers aged 6 to 36 months, resulted in increased infant and mother sleep duration, and reduced infant sleep onset latency [39]. Participant retention to the trial at follow up was very high (97 %) however a financial incentive was offered to parents who took part (ranging from $90–$175), making it difficult to determine how many parents would engage with online programs without an incentive. Other trials have demonstrated the effectiveness of online programs for a variety of purposes including educating parents on child mental health [40], increasing positive body image in adolescents [41] and treating child anxiety [42], to name a few. Online programs targeting mental health have also been useful for those in a rural or remote setting [43].

While the effectiveness of face-to-face interventions for *preventing* infant sleep and crying problems has been consistently supported, to the authors’ knowledge, there have been no trials examining whether the same advice can be effectively delivered via an online program. Such an approach may be particularly appropriate for parents who face complex and competing demands in the first few weeks postpartum. Parents may favour a resource that can be accessed at any time day or night; can be quickly and easily navigated; and that allows access to specific relevant content when the parent needs it. Initial encouragement from a MCH nurse, and/or onging participant support via email, may also be helpful in bolstering parent engagement and rentention to the program. Such strategies would be relatively easily built in to existing postpartum health services or as a feature in an online platform. With this in mind, the *Cry Baby* online program was developed. The current research aims to examine factors influencing participant engagement and retention to the Cry Baby online program, with the intention that results will inform the design of a larger efficacy trial.

### Study objectives

Our objectives are to examine:

1. whether randomisation of participants to a condition that receives additional email prompts that encourage parents to log in to the program, will result in greater use of program strategies (primary outcome) as well as greater retention to the research at follow-up (at 4 months of infant age)
2. whether parents who have the program recommended by their MCH nurse are more likely to engage with the program (as measured by program completion rates), than those who are recruited via an online advertisement for the program; and, whether they are also more likely to use the suggested strategies
3. the demographic profile of parents who choose to take part in the online program with a view to identify any over- or under-representation of parents (compared to Australian Census data where possible) based on: household income, socioeconomic status, education, language spoken and support available to parents; or child characteristics, including birth order of infant, infant age, gender, birth weight, gestation, and where the baby sleeps at night; and, to see how this differs for participants who are recruited via a popular parenting website compared to those recruited via their MCH nurse
4. the psychosocial well being of participants (specifically depression symptoms, fatigue and cognitions surrounding infant sleep in parents, and in infants, night waking behaviour, and, sleep, crying or feeding problems), with a view to identify any specific concerns that may be addressed in the development of new online programs for parents of infants

## Methods/Design

### Study design

**Fig. 1:**
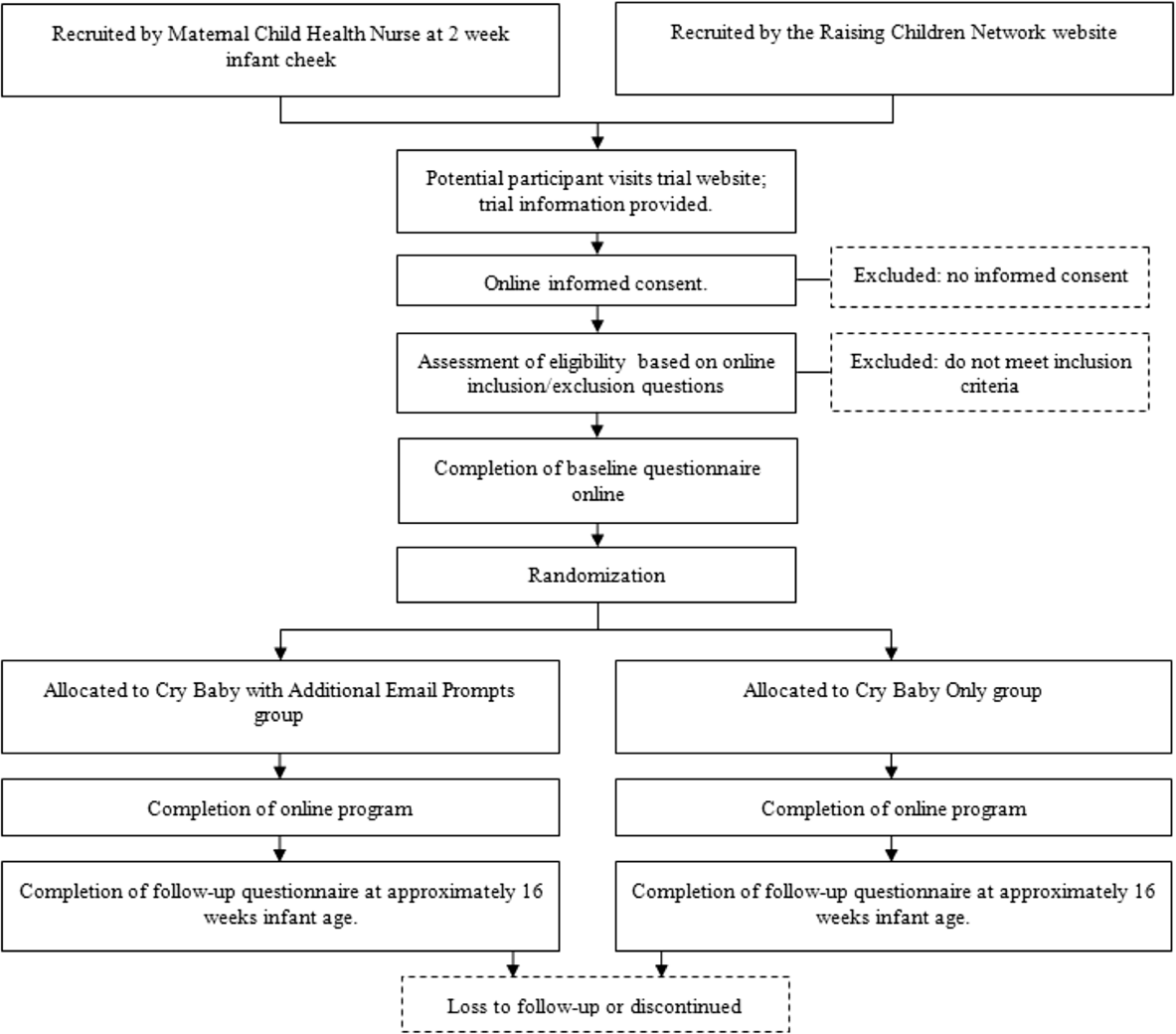
CONSORT Trial Flow Diagram

The *Cry Baby* trial is a randomised, controlled, superiority trial with two parallel groups (Fig. 1). We adhered to CONSORT guidelines in the design of the trial [44].

### Study setting and recruitment

Participants will be primary carers of infants aged 2 to 12 weeks, recruited by one of two recruitment modalities: advertising on the Raising Children Network (www.raisingchildren.net.au), or recommendation from their MCH nurse. The Raising Children Network website is a non-commercial, evidence-based, quality assured, government-funded online resource that covers a broad range of parenting topics and had over 5.7 million visits in the 2013 calendar year (personal communication). A notice will be placed on relevant pages on the Raising Children Network, as well as its social media (e.g., Facebook, Twitter). This notice will contain a link to the study website where parents can gain more information and take part in the program if desired.

Recruiting some of our sample from Australian wide online advertising and some from a specific region, introduces systematic differences to the groups. In an attempt to reduce this as much as possible, we selected the Melbourne (state of Victoria, Australia) Local Government Area (LGA) of the City of Yarra for MCH nurse recruitment, due to both its high birth rate per annum [45], and broad cultural and socioeconomic diversity [46]. All MCH nurses within this LGA (11) will invite parents of infants attending the 2 week appointment to participate in the trial. The 2 week appointment will be specifically targeted as it was suggested by the nurses that this appointment is less intensive than the in-home appointment at 7–10 days postpartum, and it is unusual for parents of infants aged less than 2 weeks to request information on infant sleep and crying so soon after birth. Recruitment will run for a 12 week period and will allow for later determination of the percentage of parents in the general community that are likely to take part in an online program if suggested by their MCH nurse (birth rate data for this region, throughout this time period will be compared to our sample). MCH nurses will provide parents with a postcard, inviting them to participate in the program. The postcard will contain brief information about the study and a link to the study website so they can gain further information about the research. Participants who indicate that they heard about the Cry Baby program via their nurse *and* via online advertising will be classified as having been recruited via their nurse since it is the effect of encouragement from a health professional that we aim to examine. The Cry Baby program is currently only available to those willing to take part in the research trial and has not been broadly advertised outside the Raising Children Network. This means it is very unlikely that parents would have the program suggested to them by other health professionals.

### Sample size and condition allocation

Power analysis using Stata 13.0 [47] was conducted in order to determine the required sample size for the study. There is no prior published data on our primary outcome (‘use of program strategies’). We have based our estimate of the required sample size on the mean total ‘strategy use’ score of the first 20 participants to be randomised to the control (Cry Baby without email prompts) condition (M = 62.53, SD = 6.04). A clinically relevant shift in the mean ‘strategy use’ score of the Cry Baby plus email prompt condition, would be half a standard deviation away from the control group mean. Based on this data, a 2 group comparative study would require a total sample size of 126 participants (63 in each arm) to have 80 % power to detect a statistically significant finding at p <.05 and a moderate effect size. Allowing for a 20 % drop out rate, we aim to recruit 152 parents.

Participants will be randomly assigned to either the *Cry Baby Only* or the *Cry Baby with Additional Email Prompts* condition, with a 1:1 allocation as per a computer generated randomisation schedule, performed as a simple randomisation. Participants will be randomised using a feature within the online survey program, which offers an online, central randomisation service. Allocation concealment will be ensured, as the service will not release the randomisation code until the participant has been recruited into the trial, and baseline measures have been completed.

### Eligibility criteria

Participants will provide informed consent online (by selecting a checkbox that appears after the Information Statement and before the baseline questionnaire), before any study procedures occur. All parents of newborn infants aged less than 12 weeks, who access www.raisingchildren.net.au or who are seen by their MCH nurse at their 2 week check in the City of Yarra will be invited to participate in the study. However, parents will be excluded from the study if they are under the age of 18 years, have a child over the age of 12 weeks at the time of enrolment into the study, have insufficient English comprehension to complete the questionnaires, have an infant born before 33 weeks gestation, or if the parent and/or infant suffer from a serious medical condition.

### Intervention

Eligible participants, who have completed baseline measures, will be randomised to the *Cry Baby Only* or the *Cry Baby with Additional Email Prompts* groups. The online *Cry Baby* program covers evidence-based information and strategies on parent self-care (e.g., taking care of your body, parent sleep, postnatal depression), infant crying (e.g., why crying is normal and healthy, why babies cry, how to cope with crying) and infant sleep (e.g., infant sleep cycles and cues, settling your baby, safe sleep conditions). All content is delivered within the online platform. The program is brief, taking no longer than an hour to complete. The program has inbuilt activities, for example, parents can move objects to make a cot a safe place for a baby to sleep, they can also ‘burst’ bubbles (using the mouse pointer) to bust common myths about baby care. There is a video showing how to wrap (swaddle) a baby for sleep, links to a coping plan for parents who are feeling overwhelmed by persistent infant crying, as well as links to local parenting support services.

Participants allocated to the *Cry Baby Only* condition will receive log in details to the *Cry Baby* program. Participants can log in or out of the program whenever they please and can move around different areas of the program according to their interests and requirements. If participants decide to complete the program in one sitting it will take approximately 1 hour to finish. The program is designed to be accessed during the first 6 to 8 weeks post-partum, as this is the time when infant crying is most frequent and the advice offered may be most useful. However, we will allow parents of infants up to 12 weeks of age to take part, as evidence indicates that infants who are very unsettled (this may continue after the usual peak in crying has subsided) are at increased risk for AHT [48]. The Cry Baby program may be useful for these parents, and gathering data from those with babies aged 8–12 weeks, will allow for later determination of the age at which parents find the program most useful.

Participants who are randomly allocated to the *Cry Baby with Additional Email Prompts* condition will receive access to the *Cry Baby* program and brief, weekly email prompts from the research team. These messages will contain evidence-based, age appropriate information on infant sleep and crying and parent self care, that is already provided within the online *Cry Baby* program. The email prompts will also direct participants back to the program for more detailed information. As above, participants will be encouraged to access the program before their infant is 6 to 8 weeks of age (when possible), and are encouraged to revisit the *Cry Baby* program as often as needed.

Participants may withdraw from the study at any time for any reason. Likewise, participants allocated to the *Cry Baby with Additional Email Prompts* condition may withdraw from receiving the additional email prompts at any time.

### Outcome measures

Our primary outcome measure will be the frequency with which participants use the strategies recommended in the program (see description of the Follow-Up Questionnaire below). This will be compared across recruitment mode (recruited via the Raising Children Network or via MCH nurse) and trial arms (Cry Baby only versus Cry Baby with Additional Email Prompts conditions).

Secondary outcome measures include: retention to the program at follow up; program completion rates (indicated by every module of the program having been accessed); participant demographic characteristics; infant characteristics; whether the participant would recommend the program to family/friends; report of infant sleeping, crying and feeding problems; average time the infant spends awake during each night waking; and, parent depressive symptoms, fatigue and cognitions surrounding infant sleep.

#### Baseline questionnaire

The participant will be asked to complete a baseline questionnaire online. This will collect demographic information about the parent (age, gender, educational attainment, employment status, country of birth, main language spoken at home, and household income) and infant (birth order, date of birth, gender, birth weight, gestation, and where the baby sleeps at night). Area-level socioeconomic disadvantage will be determined using the *Socio-economic Indexes for Areas* [46] based on participant postal code and population census data.

#### Follow-up questionnaire

A secure web link to the follow-up questionnaire will be emailed to participants when their infant is 16 weeks of age. This represents an age at which most infants ‘sleep through the night’ (i.e., achieve 5 consecutive hours of uninterrupted sleep during the night) [49].

##### Parental use of intervention

A series of questions were developed specifically for the current study. Participants will be asked how often they use the settling (8 items), safe sleep (3 items), and parent wellbeing (5 items) components of the intervention on a 5-point scale, 1 = *Never* to 5 = *All the time*. Mean scores will be computed for the three types of intervention strategies. Participants will also be asked whether they would recommend the program to friends or family, how much of the program their partner participated in (watched/listened to: none/small amount/around half/most/entire program), what was the best part of the program and how could the program be improved.

##### Caregiver support

Participants will rate the level of support or help they received from (1) their partner, (2) family and friends living elsewhere (i.e., I get enough help, I don’t get enough help, I don’t get any help, I don’t need any help), and (3) how often they feel they need support or help but can’t get it from anyone (i.e., very often, often, sometimes, never, I don’t need it) [50]. These items have previously been used in the Longitudinal Study of Australian Children [50] and in the *Baby Business* trial [3].

##### Infant behaviour

Participants will be asked whether infant daytime sleep, night-time sleep, feeding or crying behaviours have been a problem over the last 2 weeks (0 = *No*; 1 = *Yes*). If participants indicate that any of these infant behaviours are a problem, they will be asked to rate the extent on an 8-point scale, 1 = *Hardly a problem* to 7 = *Severe problem*. These items have been used in prior infant sleep research [3, 51].

Additional items will examine: the average number of times per night the participant attends to their infant (1–10+); at each attendance, the number of minutes the participant spends with their infant (<10mins – 2 h); and the average number of feeds the infant has over a 24 h period (items previously used [3]).

##### Postnatal depression

Symptoms of depression will be assessed using the Edinburgh Postnatal Depression Scale [52], a 10 item validated screening tool for PND [53, 54]. Clinically significant levels of depressive symptoms are indicated by scores ≥ 10 and ≥ 9 for mothers and fathers, respectively, in community samples.

##### Fatigue

Both the Fatigue Assessment Scale [55] (FAS) and the Fatigue Severity Scale [56, 57] (FSS) will be used to assess participants’ levels of fatigue. The FAS is a 10 item measure that will be used to assess the physical and cognitive symptoms of fatigue. Items are rated on a 5-point scale (1 = *Never* to 5 = *Always*), where higher scores indicate higher levels of fatigue. The scale has good reported psychometric properties and has been validated for use with parents [58]. The 9 item FSS assesses the intensity of fatigue and associated functional limitations caused by fatigue. Items are rated on a 7-point scale (1 = *Strongly Disagree* to 7 = *Strongly Agree*), where higher scores reflect higher levels of fatigue.

##### Parental cognition around infant sleep

Four subscales of the Maternal Cognitions about Infant Sleep Questionnaire [59] will assess participants’ cognitions about their infants’ sleep. These subscales measure limit setting (e.g., ability to resist infant demands and not be overintrusive in helping the infant fall asleep; 5 items), anger (e.g., anger, regret, and helplessness; 5 items), doubt (e.g., uncertainty regarding ability as a parent; 5 items), and safety (e.g., excessive concerns about cot death; 2 items). Items are rated on a 6-point scale, ranging from 0 = *Strongly disagree* to 5 = *Strongly agree*.

##### Online program usage

Data will be collected via the programming of the online system, recording whether the program was completed (if all program modules were accessed for a minimum of 1 min each), how many times the participant logged in to the program, and how much time in total they spent within the program.

### Procedures

The recruitment phase will run for 12 weeks in each recruitment modality. An advertisement placed on the Raising Children Network will provide interested parents with a link to the program’s online landing page. MCH nurses will provide all parents of newborn infants at the 2 week infant check with an advertising postcard containing the link to the landing page.

The landing page will provide more information describing the study, what participation involves, and a secure link to the online baseline survey where participants can read the projects’ plain language statement and complete eligibility criteria and consent. After completion of the baseline survey, participants will receive email notification of their allocation to condition (i.e., *Cry Baby* alone or *Cry Baby with Additional Prompts*). The final page of the baseline survey will provide participants with a secure link to the *Cry Baby* website, where they can register their login details and immediately access the *Cry Baby* program.

All participants will receive a welcome email which contains further information about the program and their group allocation. Participants allocated to the *Cry Baby with Additional Prompts* condition will also receive a brief, age-appropriate email prompt each week (from 2 weeks of infant age) until their infant reaches 12 weeks of age. Each email will contain a brief summary of information from the online program.

When participants’ infants are approximately 16 weeks of age, they will receive an email link to the follow up survey which has their unique ID code embedded. If participants have not completed their follow-up questionnaire after 2 weeks, a reminder email will be sent a maximum of three times over a 6 week period. At this time point, some parents will only have had access to the program for 4 weeks, and others for 14 weeks, however, we expect that if parents are going to try out strategies suggested in the program, they would do so within 4 weeks of their first access to the program. Setting the follow up time point according to infant age, rather than time since enrolment, controls for the large variability in infant development that might otherwise impact on our results.

### Data management

All data will be collected electronically using an online survey tool which provides a secure survey website (SSL encryption). Password protected data files will be utilised for participant tracking during the study and data analyses. Access to the study data will be restricted to the research team. Data will be securely stored for a period of 7 years after the completion of the study. Backup of electronic data will be performed regularly and stored securely on a password protected internal network.

### Statistical methods

A range of descriptive and multivariate data analyses will be used to assess the aims of the study. Descriptive statistics (e.g., Chi-square for categorical variables; means and standard deviations for continuous variables) will be presented for the sample demographics, number of times participants access the program, and proportion of participants completing the program. Demographic characteristics of the sample will be compared to country-wide averages obtained from the most recent Australian Census data. Characteristics of infants taking part in the research will be compared to MCH data for babies born within the same geographic area at the time of study recruitment. A mixed-design analysis of variance (split-plot ANOVA) will be conducted to compare the intervention conditions (between-subjects factor) on each of the continuous outcomes at follow-up (within-subjects factor). Effect sizes will be reported where appropriate, with 0.01, 0.06 and 0.14 as small, medium and large effect sizes for multivariate η2, while 0.2, 0.5 and 0.8 are small, medium, and large effect sizes for Cohen’s *d*. All analyses will control for child age given that time of enrolment varied between 2 and 12 weeks postpartum. Finally, intention-to-treat analysis will be conducted using Full Information Maximum Likelihood for missing follow-up data.

### Human Research Ethics Committee approval

The current study has received approval from the Parenting Research Centre’s Human Research Ethics Committee (HREC; 17/12/2012). Any modifications to the study protocol which may impact on the conduct of the study, potential benefits of the participants or may affect their safety, including changes of study objectives, study design, participant population, sample sizes, study procedures, or significant administrative aspects will require a formal amendment approved by the principal investigators and HREC.

## Discussion

*Cry Baby* is the first randomised, controlled, superiority trial to examine whether evidence based advice on managing infant sleep and crying can be effectively delivered online. This trial will establish whether involvement from a health professional and/or weekly email prompts are necessary to engage parents of newborns with online programs, and additionally, provide insight in to the demographic characteristics of parents who choose to access advice online. Our findings will inform the development, design and retention strategies of other trials of online programs.

## Abbreviations

AHT: Abusive Head Trauma
FAS: Fatigue Assessment Scale
FSS: Fatigue Severity Scale
HREC: Human Research Ethics Committee
MCH: Maternal and Child Health
PND: postnatal depression
SSL: secure sockets layer
